# Feasibility study on the adoption of the WHO safe childbirth checklist by front-line healthcare providers and managers in Burkina Faso and Côte d’Ivoire

**DOI:** 10.1186/s40814-020-00691-1

**Published:** 2020-10-06

**Authors:** Kadidiatou Raissa Kourouma, Wambi Maurice Evariste Yaméogo, Daouda Doukouré, Marie Laurette Agbré Yacé, Akoua Tano Kamelan, Soltié Aminata Coulibaly-Koné, Tiéba Millogo, Séni Kouanda

**Affiliations:** 1 Institut National de Santé Publique/Cellule de Recherche en Santé de la Reproduction, Abidjan, Côte d’Ivoire; 2 Institut Africain de Santé Publique/Institut de Recherche en Sciences de la Santé, Ouagadougou, Burkina Faso

**Keywords:** Feasibility study, WHO safe childbirth checklist tool, Maternal and newborn care, Côte d’Ivoire, Burkina Faso

## Abstract

**Background:**

The World Health Organization Safe Childbirth Checklist tool was specifically designed for developing countries such as sub-Saharan African countries, to ensure safety and security of the couple mother and newborn around the time of childbirth. However, the implementation of the Safe Childbirth Checklist tool requires a good knowledge of the context setting to face challenges. Our study objectives were (1) to assess the acceptability of the WHO SCC tool and (2) to identify conditions and strategies for a better introduction and use of the WHO SSC tool.

**Methods:**

This was a pilot multi-country study conducted from January to March 2019 in Burkina Faso and Côte d’Ivoire, respectively, in the health regions of central-North and Agnéby-Tiassa-Mé. In each health region, 5 health facilities of different levels within the health system pyramid were selected through a purposive sampling. The study was conducted in 2 phases: 38 healthcare providers and 15 managers were first trained to use the Safe Childbirth Checklist tool; secondly, the trained providers were allowed to use the tool in real-life conditions for 2 weeks. Then, semi-structured interviews were conducted among healthcare providers and managers. The topics covered by the interview guides were acceptability of the tool, barriers and facilitators to its use, as well as strategies for better introduction and use within the healthcare system. Analysis was carried out using the Nvivo 12 software.

**Results:**

Respondents reported an overall good acceptance of using the tool. However, they suggested minor content adaptation. The design of the tool and increased workload were the main barriers to its use. Potential facilitators to its introduction were managers’ commitment, healthcare providers’ motivation, and the availability of supplies. The best strategies for optimal use were its attachment to existing tool such as partograph or/and its display in the maternity ward.

**Conclusions:**

The findings showed that the implementation of the Safe Childbirth Checklist tool is acceptable in Burkina Faso and Côte d’Ivoire. These findings are important and will help to design a trial aiming at assessing the effectiveness of the tool WHO SCC tool in these two countries.

## Key messages regarding acceptability

- The key findings are a positive attitude toward the WHO SCC tool and an overall good acceptance, and barriers and facilitators to its use are best known as well as effective strategies for its introduction and optimal use within the healthcare system.
- The implementation of the WHO SCC tool is possible in the health facilities of Burkina Faso and Côte d’Ivoire. Managers’ commitment is a key for success. Minor content adaptations must also be carried out.
- Whether the healthcare providers will be willing to use the WHO SCC tool was uncertain due to concerns about perceptions of increased workload as it was observed with the introduction of other patient management tools. It was also unclear which factors will facilitate or hinder the utilization of the tool.

## Background

There is a need to ensure women and newborn safety and security during delivery all over the world especially in developing countries. Indeed, according to WHO, more than 130 million births occur worldwide each year. Of these births, an estimated 30,300 result in mother’s death, 2.6 million in stillbirth, and another 2.7 million in a newborn death within the first 28 days of birth [1]. The majority of these deaths occur at the time of the delivery or within the first 24 h after delivery. Concerning developing countries, where the reduction of maternal and newborn morbidity and mortality rates remains a major challenge, maternal deaths account for approximately 99% (302,000) of the global maternal deaths in 2015, with sub-Saharan Africa alone accounting for roughly 66% (201,000) [2].

The WHO Safe Childbirth Checklist (WHO SCC) tool was specifically designed for these developing countries such as sub-Saharan African (SSA) countries, where resources are often limited for the successful implementation of heavy interventions. Indeed, even though the proportion of births attended by skilled healthcare providers continues to increase [3], maternal and neonatal morbidity and mortality rates remain at high levels that are unacceptable and stagnant [4]. Poor quality of care during institutional births is a major contributing factor to preventable maternal and newborn harm, death, and significant disability in developing countries [5]. Poor quality of care at the time of delivery and in the immediate postpartum can be explained by the overload of work, lack of human resources, inadequate training, and motivation of healthcare providers. All these factors lead to the development of more routine practices than systematized care made of good practices recognized as effective for both mother and newborn during childbirth and immediate postpartum period.

The use of the WHO SCC tool strategy during delivery and immediate postpartum is a relatively new practice that has the potential to prevent and/or adequately manage the main causes of morbidity and mortality in the pair mother-newborn. The tool was piloted and approved for use in delivery room in some countries in Africa (Rwanda, Namibia) Asia (Sri Lanka) and Latin America (Brazil) [6–11]. To our best knowledge, no West African countries were part of the pilot studies. The objectives of our study were (1) to assess the acceptability of the WHO SCC tool and (2) to identify conditions and strategies for a better introduction and use of the WHO SSC tool. This study was also carried out in order to inform the design of a clinical trial aiming at assessing the effectiveness of the tool in reducing poor childbirth outcomes in Burkina Faso and Côte d’Ivoire.

## Methods

### Conceptual framework

Feasibility concept is used in the assessment of health interventions, according to several definitions and approaches, and address different dimensions of an intervention such as acceptability, demand, adaptation, expansion, integration, and limited efficacy [12–15].

In this paper, the feasibility study aims to determine whether the WHO SCC tool is appropriate for further testing. Among the several dimensions on feasibility study, this study is particularly interested in the analysis of acceptability and integration [12].

Acceptability aims to answer the question: to what extent is an intervention (WHO SCC tool) considered appropriate, satisfactory, or attractive by beneficiaries or program implementers? Among the different dimensions of acceptability analysis, in this study we analyze perceived appropriateness (utility, content and form of the tool).

As for the integration dimension, it questions the elements of convergence or divergence of an intervention with an existing system. The analysis focuses here on the barriers and factors that facilitate the use of the tool, as well as the conditions and strategies for its introduction and use into the healthcare system.

The analysis is based on the health care providers’ experiences with the WHO SCC tool and local managers’ perceptions. The analysis also takes into account the perceived anticipated conditions and strategies for its introduction and use within the healthcare system [13, 16].

### Study setting

This multi-country study was conducted from January to March 2019 in Burkina Faso (BF) and Côte d’Ivoire (CI), respectively, in the health regions of central-North and Agnéby-Tiassa-Mé. These two health regions have similarities in the healthcare delivery systems with a regional hospital which is the highest level of reference. The central-North and the Agnéby-Tiassa-Mé health regions include, respectively, 130 and 131 Primary Health Care Facilities that have as direct referral centers: health district hospitals.

In each health region, five health facilities of different levels of the health pyramid were selected to be included in the study. In Burkina Faso, the health facilities selected were the Centre Hospitalier Régional de Kaya (CHR Kaya), Centre Médical Urbain de Kaya (CM Kaya), Centre medical de Tougouri (CM Tougouri), Centre Rural de Tafogo (CSPS Tafago), and Centre Rural de Dabonsnoré (CSPS Dabonsnoré). In Côte d’Ivoire, the study was conducted in the Centre Hospitalier Régional d’Agboville (CHR Agboville), Hôpital Général d’Akoupé (HG Akoupé), Centre de Santé Rural d’Assangbadji (CSR Assangbadji), Centre de Santé Urbain de Bacon (CSU Bacon), and Centre de Santé Urbain de Bécouefin (CSU Bécouefin). Apart from the regional hospitals that were purposively sampled, the other health facilities were chosen using a random sampling.

### Study design

We carried out a pilot study. The first phase consisted in the introduction of the tool to the front-line healthcare providers working at the maternity ward and local administrative including managers of the maternity unit, through training in each health facility selected. The training focused on the history, the purpose of the WHO SCC tool, its organization, and its content as well as its use.

The second phase consisted in the testing of WHO SCC tool by the trained healthcare providers under real-life conditions for 2 weeks. For this phase, two formats of the tool were given to the healthcare providers for the test. The first format was the individual WHO SCC tool on A3 sheet and for the second one, the WHO SCC tool attached to the partograph.

### Study population and sampling

The population study was composed of 67 (CI = 31 and BF = 36) healthcare providers and 15 (CI = 9 and BF = 6) managers that have been previously trained on the use of to the WHO SCC tool. After this training, the healthcare providers had 2 weeks to test the tool on a sample of birth events. Among the trained healthcare providers, 41 healthcare providers (CI = 19 and BF = 22) tested the tool in real-life conditions, and from those who tested the tool, thirty-eight (38) were interviewed (CI = 19 and BF = 19). Regarding managers, fifteen (15) were interviewed (CI = 9 and BF = 6).

### Data collection

Data were collected after the 2 weeks of testing among healthcare providers that effectively tested the tool and local managers. Qualitative data were collected through semi-structured interviews. Two interview guides were designed respectively for the healthcare providers and the managers.

The healthcare providers’ guide was designed around topics based on the conceptual framework: acceptability (experience with using the tool, perception of the tool, content and utility), strategies for better introduction and use, and barriers and facilitators to WHO SCC tool introduction and use. The guide consisted of forty questions.

Managers’ guide was made up of fourteen questions and was designed around the same topics.

In each country, data were collected by two data collectors with a master’s degree in sociology. They received 1-day training on general survey procedures and the content of the interview guides.

Participants were invited by the researchers to an individual one to one interview at the facility. Prior to interview, all participants were asked to provide written consent after a further opportunity to have their questions answered. All participants agreed to have their interview recorded.

Interviews were audio-recorded using a dictaphone and lasted on average 35 min (range 30–45 min). Saturation was achieved to the extent that we interviewed practically all of the healthcare providers who used the tool and all the managers.

#### Data analysis

The interviews were transcribed and then typed into the Microsoft Word software. A framework analysis was carried out using NVivo 12 software. Following the component of the framework presented above, we developed a codebook to describe the themes and sub themes and used it to code the transcripts. The results of the coding were synthesized in matrices according to themes (acceptability of the WHO SCC tool, barriers and facilitators of the WHO SCC tool use and introduction, conditions and strategies for a better introduction and optimal use)

## Results

### Participant characteristics

The study included 53 participants (25 participants in Burkina Faso and 28 in Côte d’Ivoire). In Burkina Faso, the sample was composed of 2 obstetricians, 5 assistant midwife, 12 midwives, and 6 managers. The average age of the healthcare providers was 39.2 ± 5.3 years old.

In Côte d’Ivoire, the sample consisted essentially of midwives (19) with an average age of 36.4 ± 5.5 years old and an average years’ experience of 4.7 ± 3.1.

In both countries, the majority of the sample was composed of women. Background characteristics of the respondents are presented in Table 1.

**Table 1:** Background characteristics of the respondents

	Burkina Faso				Cote d'Ivoire			
	Total number	Age (mean)	years' experience mean	sex ratio	Total number	Age (mean)	years' experience (mean)	sex ratio
**Local Manager**	6	43	9.7±5.5	2	9	42.7±8.2	12.5±9.7	2
**Healthcare providers**	19	39.2±5.3	10.8±5.1	0.06	19	36.4±5.5	4.7±3.1	0
*Assistant midwife*	*5*	39±4.4	11.6±3.8	0	─	─	─	─
*Midwives*	*12*	39.1±4.8	10.8±5.3	0	19	36.4±5.5	4.7±3.1	0
*Obstetricians*	*2*	12	9.9	1	─	─	─	─

For the next results, healthcare provider will be abbreviated “HP” and manager “M”.

### Acceptability of the WHO SCC tool

The subthemes related to the acceptability were perceived utility, the perceived simplicity, and the content of the tool.

#### Perceived utility of the WHO SCC tool

All respondents in both countries agreed that the WHO SCC tool is a very helpful tool to improve medical practices and thus minimizing errors.

Some respondents perceived the WHO SCC tool as a complementary tool to existing tool such as partograph, childbirth register.

In that regard, one respondent in Burkina Faso said:

The WHO SCC tool is useful, because it is a document that supports the partograph. (HP16, Burkina Faso)

In Côte d’Ivoire, one respondent had the same perception:

It is useful because it provides guidance in relation to a problem you encounter while monitoring your work ……when you refer to your checklist that gives you a solution. Even though, we used to perform certain acts before, with the WHO SCC tool it goes even faster. (HP26, Côte d’Ivoire)

For other respondents mainly in Burkina Faso, the WHO SCC tool is like a decision aid. It allows detecting abnormalities in time and directing on the treatment to perform. One respondent explained it in these terms:

It’s the same thing as the partograph, it’s about the parturient’s monitoring. If we manage to monitor with the WHO SCC tool, it will allow us to make the decisions in time and avoid complications.’ (HP9, Burkina Faso)

#### Perceived simplicity of the WHO SCC tool

In both countries, the WHO SCC tool was perceived as an easy-to-use tool. Respondents emphasized above all the fact that the tool does not ask for information to be filled in, but simple check-boxes as pointed. For one healthcare provider in Côte d’Ivoire:

It’s not complicated! That’s what I said earlier. It is simply a check. Okay! So it’s not difficult, short sentences, very explicit. This is not difficult to understand. (HP18, Côte d’Ivoire)

#### Content acceptability

There was a consensus that the font size on the tool is too small. Some respondents who shared this perception also pointed out that this aspect could make it difficult to use the tool. One respondent expressed it as follows:

You have to improve the font; the letters are too small. You have to increase it a little bit. (M15, Côte d’Ivoire)

Another respondent confirmed that statement:

Regarding the format, it is too condensed and not readable, the font is very small. With rooms that are not lit as it should, it will be difficult to read at night. (HP4, Burkina Faso)

Besides, there was conflicting opinion about the length of the WHO SCC tool.

Indeed, all the respondents in Côte d’Ivoire found that the WHO SCC tool is not long, as stated by this respondent:

At a glance, the WHO SCC tool looks long. But when you start reading, it’s not long, but there are certain details they have given. It is fine, it’s okay! (HP25, Côte d’Ivoire)

However, in Burkina Faso, some respondents judged the WHO SCC tool to be too detailed and also found that it takes into account all the elements that are already contained in the patient’s record. This perception is illustrated by the following quotation:

The WHO SCC tool is long and it includes all the elements of the patient’s file. It is a kind of summary. If they can reduce the list of items, it will be better because it is like a rework of the patient’s file (HP16, Burkina Faso)

### Barriers and facilitators to the WHO SCC use

#### Barriers to WHO SCC use

Respondents were asked about the barriers and facilitators of the WHOSCC tool use when testing it. From the interview two major barriers emerged: the increased workload and the design of the WHO SCC tool.

##### Increased workload

In both countries, respondents mentioned an increased workload when using the WHO SCC tool especially when they received several parturients at the same time. This situation is described below:

The use of the tool was very difficult for me especially when I received a lot of women, it increases the workload…I had to fill the tool and take care to the women at the same time … that was not easy at all!!!! (HP3, Burkina Faso)

##### The design of the WHO SCC tool

As regards the WHO SCC tool, the design of the tool included font size emerged as major barriers for some respondents, as stated by this healthcare provider:

During the test, I could notice it was better that the WHO SCC was embedded in the partograph. When it is in the form of a loose leaf, it is easy to forget to use it. Moreover the font size is too small which made it a bit difficult to use. (HP9, Burkina Faso)

#### Facilitators to WHO SCC use

The most cited facilitators were the fact that there are no new acts to be performed and the simplicity of the tool, as illustrated below by one respondent:

At first sight, the WHO SCC tool seems to be long, but I see that it is simply for checking the acts that need to be performed during delivery .... It’s like a summary of our medical record ... It’s simple since we are only asked to check. (HP5, Burkina Faso)

### Conditions and facilitators to WHO SCC introduction

The major facilitators related identified were managers’ commitment, healthcare providers’ motivation, and availability of supplies.

#### Managers’ commitment

In both countries, all respondents emphasized the importance of the managers’ commitment which can positively impact on the introduction of the WHO SCC tool. Managers’ commitment must also be done through supervision. This point of view is illustrated by one manager in Burkina Faso:

It must be a commitment at the institutional level, for it to be a success; otherwise the process will be blocked. We have to show to the practitioners that the WHO SCC tool is important by doing some supervision. Otherwise it will not work in the field. (M3, Burkina Faso)

#### Healthcare providers’ motivation

The motivation of healthcare providers was also cited by some respondents as a facilitator of the introduction. According to them, this motivation can be in different forms: sensitization, training, and financial incentive. The majority of managers and healthcare providers stated that is necessary to work on mentalities through creating awareness and training to show the validity of the WHO SCC tool as a job aid and not as an extra work. Besides, in Burkina Faso as well as in Côte d’Ivoire, some respondents declared that healthcare providers could be motivate through financial incentive. This financial incentive linked to the utilization of the WHO SCC tool would be based on performance.

One manager in Burkina Faso stated:

It is important that everyone is at the same level of understanding of the WHO SCC tool. Training and sensitization are important so that everyone is impregnated at the same time before disseminating the WHO SCC tool. (M6, Burkina Faso)

The same concern was expressed in Côte d’Ivoire by one manager:

You have to financially motivate the staff. For motivation, frankly, I like the performance-based financing model. If this model is applied to the WHO SCC tool it will be a success. (M14, Côte d’Ivoire)

#### The availability of supplies

In both countries, according to respondents, the sustainable use of the WHO SCC tool requires the availability of the WHO SCC tool itself as well as the supplies, as stated by one of them:

To facilitate the use of the WHO SCC tool, the tool itself must be available as well as the supplies. We get used to the tool and then we are told that it is out of order or that this or that input is missing. If we decide to implement the WHO SCC tool, the health structures must be equipped for it to be sustainable. (HP27, Côte d’Ivoire)

This statement was confirmed by another respondent in Burkina Faso:

We must think about equipping the maternity. If the material is not available, it is not obvious. We will sometimes check not as if the act had not been done while it is the missing equipment. It is necessary that the drugs are available especially the magnesium sulphate ... Even in the delivery room, it misses things. The equipment for medical reanimation, we do not have all that. (HP17, Burkina Faso)

### Strategies for introduction and optimal use of the WHO SCC tool

**Fig. 1:**
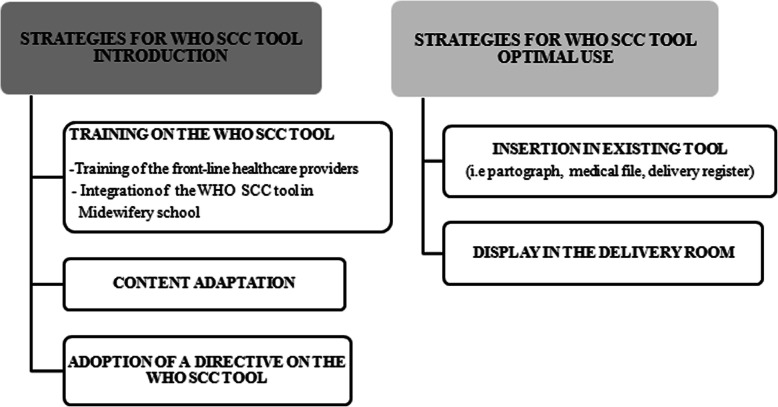
Strategies for WHO SCC tool introduction and optimal use according to respondents

Participants in the study were asked about strategies concerning the WHO SCC tool introduction and optimal use (Fig. 1).

#### Strategies for WHO SCC tool introduction

Concerning the introduction of the WHO SCC tool, three suggestions emerged from the interviews: training on the WHO SCC tool, content adaptation, and the adoption of a directive regarding the use of the WHO SCC tool.

##### Training on the WHO SCC tool

***Training of front-line healthcare providers*** According to respondents, training will allow them to remove any ambiguity from the introduction of the WHO SCC tool and put all the providers at the same level of knowledge. This was expressed by this manager in these terms:

If the healthcare provider did not receive training on the WHO SCC tool, if we do not warn that there is a new tool that must be respected in our different register, it will also create an hindrance to fill the WHO SCC tool. If everyone is aware and everyone knows how to fill it and everyone has been well trained, you will see that we will fill it correctly. (M10, Côte d’Ivoire)

***The insertion of the WHO SCC tool in midwifery training school curriculum*** Moreover for some respondents, to introduce the tool, it is necessary to insert training on the tool in midwifery school curriculum so that they are impregnated from the beginning.

In alignment with this statement, one respondent said:

I propose to introduce the WHO SCC tool from the base, in midwifery school training curriculum, so that new generations will show skills with the tool. It avoids a lot of things ... You know, often the agents are a bit oblivious. If they don’t learn at school, when they go out and see the tool in the field, some of them will say: I have not been trained on this tool so I can’t use it ... it got complicated. You have to introduce it at school... No one would say that he was not trained. (HP9, Burkina Faso)

##### Content adaptation

Respondents suggested some content adaptation. For instance, as for the phase “soon after birth”, seventeen respondents in Burkina Faso suggested reversing the order of the items by making the acts checked for the child first. This suggestion is illustrated below:

A priori when the woman gives birth, we take care of the child first to ensure his condition, see if he can adapt to the extra-uterine life and if this is not the case we try to revive it or transfer it ...... After, we take care of the mother. (HP13, Burkina Faso)

In Côte d’Ivoire, some respondents suggested adding a new item to check the insertion of the Bakri postpartum balloon catheter when the woman is bleeding after childbirth as stated by one of the respondents: “It is necessary to immediately put the catheter to the woman in case of hemorrhage. Of 5 women who bleed when you put the catheter, the bleeding stops in 90% of cases.” (HP25, Cote d’Ivoire).

##### The adoption of a directive regarding the use of the WHO SCC tool

For the majority of the respondents in Côte d’Ivoire, to implement the WHO SCC tool it is crucial to do it in a formal framework. This point of view is illustrated below by one manager:

There is a need to have a directive related to the utilization of the WHO SCC tool. It will allow us to pressure the healthcare providers to use it. (M11, Côte d’Ivoire)

#### Strategies for an optimal use of the WHO SCC tool

As regards strategies for an optimal use of the tool, two suggestions arose: its insertion in existing tool and its display in the delivery room.

##### Insertion in existing tool

In both countries, most of the respondents emphasized the importance of the WHO SCC tool location. For the vast majority of them the best strategy to implement the WHO SCC tool is to embed it into the conventional registers such as the delivery register. One respondent said:

It must be inserted in the register file, in other words, the conventional registers. Given the fact that the practitioner knows that he is required to inform items there, it will promote the adoption, the appropriation of this document. (M11, Côte d’Ivoire)

##### Display in the delivery room

Besides, some respondents suggested that the WHO SCC tool should be displayed in the delivery room, as stated by one of them:

You have to make the WHO SCC tool in large format and display it in the delivery room; it will allow everyone to see it. If it’s in the records, it will not be easy, but if it’s displayed in great character, you only have to look at the steps. (HP8, Burkina Faso)

## Discussion

The WHO SCC tool was developed by World Health Organization to improve adherence to life saving practices in the intra- and immediate postpartum period. The aim of this study was to evaluate the tool for feasibility among healthcare providers and local administrative including managers in Burkina Faso and Côte d’Ivoire. This data enable researchers to understand the acceptability of the WHO SCC tool, to identify barriers and facilitators to its introduction and use and, as well as strategies for introduction and optimal use of the WHO SCC tool.

### Acceptability of the WHO SCC tool

The acceptance and implementation of innovative practices different from those practiced daily are in fact a change of behavior, and are recognized as a challenge [7, 10]. In our study, the acceptability of the WHO SCC tool was measured with healthcare providers and managers in health facilities of different levels of reference. The study findings indicated that both healthcare providers and managers had a good perception of the WHO SCC tool, and their attitudes toward acceptance to use the WHO SCC tool were positive. These findings are consistent with other studies performed in countries where the WHO SCC tool has been implemented, showing satisfactory attitudes toward acceptance [6, 7, 9, 11]. This positive attitude toward the WHO SCC tool in our findings shows a willingness of the healthcare providers to use the tool.

Additionally, the WHO SCC tool is perceived by the respondents as a complementary tool to existing tool such as partograph, as a decision aid and also as a very useful and simple tool for improving practices and minimizing errors.

### Facilitators and barriers of the WHO SCC tool use and introduction

Identification of facilitators and barriers is fundamental to achieve a better uptake of interventions and to improve the implementation of clinical practice strategies [17, 18]. With regard to the use of the tool during the test, the findings showed that increased workload and the design of the WHO SCC tool were the main barriers to its greater use. These findings are consistent with other studies conducted in countries where the WHO SCC tool has been implemented [7, 8]. However, in the study conducted by Perry et al., the design of the tool was found good by the end users and the implementation team [8].

To overcome these hindrances for an optimal use of the tool, it is worthwhile to adapt the design of the WHO SCC tool for instance by increasing the font size, and also to deal with other factors that could reinforce increased workload such as understaffing.

Regarding the introduction of the WHO SCC tool, managers’ commitment, healthcare providers’ motivation, and the availability of supplies were cited as facilitators. Managers’ commitment is one of the keys to success for the delivery of good health services and should be done through an effective leadership. Indeed, managers with effective leadership can motivate their staff and are able to negotiate for supplies, resources, and other supports to create good working environment. In many studies conducted to evaluate adherence to the WHO SCC tool, the authors concluded that the strategy adopted that included the commitment of managers and authorities can contribute to improving adhesion and engagement of healthcare providers [6, 8, 9].

For the respondents, motivation through supervision, training of financial incentive is important to adhere and use the WHO SCC tool. Indeed, healthcare providers’ supervision and training can increase job satisfaction and motivation leading to a better appropriation and use of the WHO SCC tool. In many sites, senior staff were trained to mentor, supervise, and encourage others to use the WHO SCC tool and this is considered key success [8, 9, 19].Concerning financial incentive, in a context where African countries are increasingly engaging in performance-based financing to improve health services quality, this could be an effective way to ensure the sustainable use of the WHO SCC tool. However, providing financial incentive could lead to negative impacts, for instance healthcare providers may only focus on the WHO SCC tool and reduced their effort in other services that are not rewarded.

Financial incentive, supervision, and training cannot alone permit the appropriation and use of the tool. Indeed, the availability of supplies is also important to facilitate the use of the WHO SCC tool. A shortage in medicines and other supplies may affect healthcare providers’ willingness to use the WHO SCC tool. In India where the tool has been implemented; coaching-based intervention (Better Birth Program) on availability and procurement of essential childbirth related supplies was performed showing a positive impact. This shows once again the importance of effective leadership and coaching in the implementation of the WHO SCC tool.

### Conditions and strategies for the WHO SCC tool introduction and use in the health system

In our study findings, regarding the WHO SCC tool introduction strategies, respondents proposed to carry out training on the tool and content adaptations to adopt a directive on the WHO SCC tool. Indeed, an adaptation of the WHO SCC tool is important to ensure consistency with local guidelines and to prompt willingness among end users to adopt the WHO SCC tool [20] as it was done in most countries that have implemented the WHO SCC tool [6–9, 11, 21–24]. Hence, in our study, an initial modification of the WHO SCC tool based on local context was suggested by respondents. Minor adaptations that were suggested included the introduction of an item on the Bakri ballon. This suggestion on the Bakri ballon can be explained by the fact that the Bakri Balloon is recommended as a treatment line for postpartum hemorrhage unresponsive to uterotonics [25]. Indeed, postpartum hemorrhage is one of the leading causes of maternal death in areas where essential care and skilled health attendants are limited such as African countries.

As regards directives, they have the benefit to provide clear guidance to healthcare providers and regulate their work. Directives also aim to improve the quality of care and to promote patient safety by presenting the current evidence base and translating it into clinical practice. If a directive on the WHO SCC tool is adopted, it will be mandatory to use the WHO SCC tool and the healthcare providers will comply. However, it has been proved that adherence to guidelines declines. In addition, the publication and dissemination of guidelines do not, on its own, automatically result in their use [18].

Concerning training of the healthcare providers on the use of the WHO SCC tool and Essential Birth Practices, performing refresher training could help to tackle the lack of training. The integration of WHO SCC tool in midwifery school training curriculum was also recommended by the respondents as a strategy of introduction. Concerning this suggestion, since the WHO SCC tool is a memory aid at their disposal, it would be interesting to have a focus on essential birth practices contained in the tool in training curriculum. This will have the advantage of impregnating the students on the fundamentals for a better usage of the WHO SCC tool, creating awareness and allowing them to become familiar with the essential birth practices of the tool even before their assignment in health structures. Another advantage is harmonization of the knowledge so that once in the field, it will be enough to conduct refresher training. This will help to anticipate a shortage in practices due to turn over.

The way in which the WHO SCC tool is introduced for usage to healthcare providers is important. Our study findings reported two strategies for an optimal use of the WHO SCC tool: the insertion of the WHO SCC tool in existing tools and its display in the delivery room. In a context where providers have the impression of using a lot of tools, the WHO SCC tool should not be presented as a new tool, but rather as a complementary tool to existing tools. Thus, in our findings, the majority of the respondents were unanimous on the fact that the WHO SCC tool will be difficult to use if it remains isolated and that it should therefore be embedded to an existing tool such as partograph, medical file, or register of consultation. In the majority of the countries where the WHO SCC tool has been implemented, this one was attached to mothers’ clinical notes, charts from labor to discharge [6, 7, 10, 21]. As regards partograph, it remains a useful tool and a very good indicator of maternal and newborn quality of care. Its use helps to improve maternal and fetal outcomes. Even though healthcare providers have shown a good acceptance of the partograph, there is evidence that it is not being used as anticipated in practice; hence, it is failing to reach its potential in improving outcomes [26]. Attaching the partograph to the WHO SCC tool could help to improve maternal and newborn outcomes.

The display of the WHO SCC tool in the delivery ward if it has the advantage of being practical, the provider not to hold a tool, it could suffer the fate of the other tools posted in rooms whose degraded state raise doubt over their use. In addition, placing the WHO SCC tool in large format in the delivery room would not allow using it once the woman leaves the delivery room. This option implies that the tool is posted in all rooms where the woman goes before leaving the facility. In a study conducted in Rwanda, the WHO SCC tool posters were also posted on the wall around the delivery ward to remind clinicians of the importance of Essential Birth Practices [11].

### Study strength and limitations

Evidence generated in this study will help to the elaboration of a trial protocol related to the effectiveness of the WHO SCC tool in Burkina Faso and Côte d’Ivoire. This study also contributes to filling a knowledge gap in these contexts setting. Thus, the process of implementing the WHO SCC tool is a complex social intervention that requires changes in the end user’s perspective regarding the perception about WHO SCC tool and mother and child safety but also effective leadership. This process should be based on the elements that can facilitate, but also consider the organizational and material constraints of health facilities, as well as the appropriate strategies for the WHO SCC tool introduction and use.

However, this study has few limitations. All participants trained in the use of the WHO SCC tool could not use the WHO SCC tool. Indeed, in regional and general hospital, a staff rotation system did not allow some trained midwives to use the tool as they were delegated to other tasks such as family planning and immunization.

## Conclusions

This study addresses a relevant issue which is the feasibility of the implementation of the WHOSCC tool to reduce maternal and perinatal death in Burkina Faso and Côte d’Ivoire. The findings revealed that the implementation of the WHO SCC tool is feasible in the two selected countries. Healthcare providers showed a good acceptance toward the tool and minor content adaptation must also be carried out. To be most successful, the implementation should be tailored to fit the needs of the healthcare providers within their working environment and also take into account the barriers and drivers identified.

## Data Availability

Séni Kouanda, senikouanda@gmail.com.The data presented in this study are from the WHO Safe Childbirth Checklist project conducted in Burkina Faso and Côte d’Ivoire. Access will be granted only after careful and due consideration of the compliance with the ethics requirements and the data policy of the Institut de Recherche en Sciences de la Santé.

## Abbreviations

HP: Healthcare provider
HRP: Human Reproductive program
M: Manager
SSA: Sub-Saharan Africa
WHO SCC: World Health Organization Safe Childbirth Checklist
CI: Côte d’Ivoire
BF: Burkina Faso
